# Dissolution of a fibrous peptide by terahertz free electron laser

**DOI:** 10.1038/s41598-019-47011-z

**Published:** 2019-07-23

**Authors:** Takayasu Kawasaki, Koichi Tsukiyama, Akinori Irizawa

**Affiliations:** 1 IR Free Electron Laser Research Center, Tokyo University of Science, 2641 Yamazaki, Noda, Chiba 278-8510 Japan; 2 The Institute of Scientific and Industrial Research, Osaka University, Mihogaoka 8-1, Ibaraki, Osaka 567-0047 Japan

**Keywords:** Photobiology, Free-electron lasers, Terahertz optics, Peptides, Infrared spectroscopy

## Abstract

Fibrous peptides such as amyloid fibrils have various roles in biological system, e.g., as causal factor of serious amyloidosis in human and as functional regulator of cell formation in bacteria and eukaryotes. In addition, the fiber-type format is promising as biocompatible scaffold. Therefore, the dissolution method of peptide fibril is potentially useful at many scenes in medical and material fields: as reductive way of pathogenic amyloid, as modification technique of cell structure, and as fabrication tool of biomaterials. However, the fibril structure is generally difficult to be dissociated due to its rigid stacked conformation. Here, we propose a physical engineering technology using terahertz free electron laser (FEL) at far-infrared wavelengths from 70 to 80 μm. Infrared microscopy analysis of the irradiated fibril of calcitonin peptide as a model showed that β-sheet was decreased, and α-helix, turn, and others were increased, compared to those of the fibril before the FEL irradiation. Interestingly, the dissociative effect by the far-infrared laser was remarkable than that by the mid-infrared laser tuned to 6.1 μm that corresponds to amide I. In addition, simple heating at 363 K deformed the fibril state but increased the amount of β-sheet, which was contrast with the action by the FEL, and scanning-electron microscopy and Congo-red staining revealed that the fibril was collapsed power-dependently within a range from 25 to 900 mJ energies supplied with the FEL at 74 μm. It can be considered that irradiation of intense terahertz wave can dissociate fibrous conformation of peptide with little influence of thermal effect.

## Introduction

Self-assembly of proteins and peptides have various roles in expression of biological function. As typical example, amyloid fibril is a kind of those aggregates, and it is closely associated with serious diseases such as amyloidosis. Much biochemical researches for discovery of pharmaceutical drugs targeting amyloid fibrils have been extensively conducted lately^1–3^. In addition, some species of amyloid fibrils work as functional regulators of gene expression in a wide range of biological systems from bacteria to eukaryotes including mammals, and those functional amyloids are rather critical factor for maintaining the life activity^4^. A structural characteristic of fibrous peptide is β-sheet stacked conformation, and its hydrophobic structure is thermodynamically stable under physiological conditions. The stacking structure is a common characteristic in almost kinds of amyloid fibrils even though the amino acid sequences are different. Another remarkable feature of the peptide fibril is such a tough fiber-like format. The natural products and the artificial molecules which are constructed by the regular fiber structure can be employed as reliable scaffold for biocompatible materials^5,6^. Therefore, development of structural deformation method of the fibrous aggregate will be expected to lead to processing system of solid biomaterials, reduction of pathogenic amyloid, and regulation of biological function associated with peptide fibrils. Nonetheless, the fibrous construct is so rigid and hardly dissolved in water unless being exposed to denaturation reagents and organic solvents.

**Figure 1:**
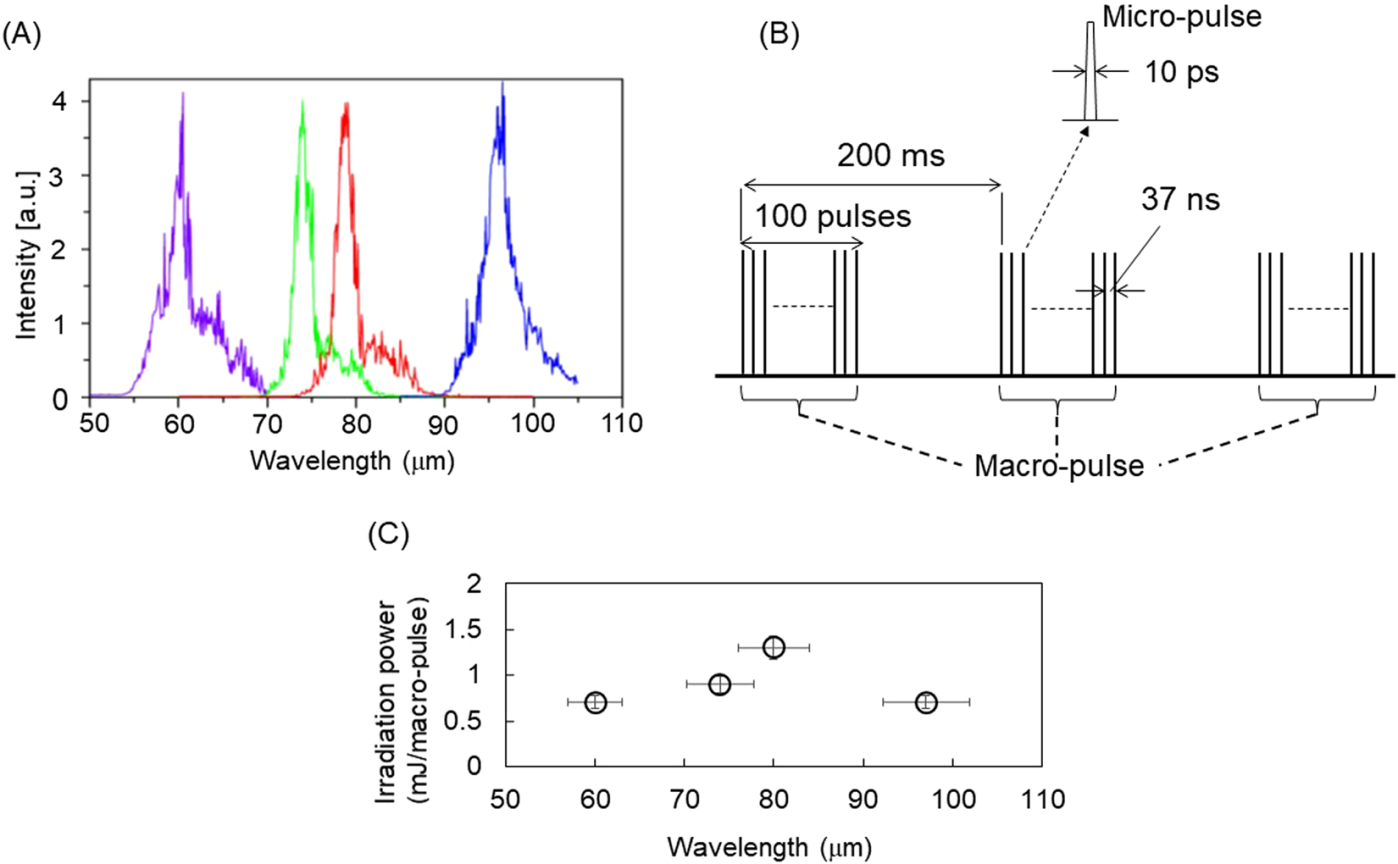
Oscillation parameters of FIR-FEL. (**A**) FEL spectra at various far-infrared wavelengths (60, 74, 80, and 97 μm). (**B**) Pulsed time structure. (**C**) Irradiation energy per macro-pulse at each wavelength.

In material and biomedical fields, physical techniques using lasers and strong electromagnetic waves are often employed for fabrication, structural alteration, and functional control of chemical and biological substances^7–9^. Particularly, infrared free electron laser (FEL) enables us to investigate novel laser-driven phenomena in various research fields, due to its high photon density in a picosecond time scale as well as wide wavelength tunability in the infrared region^10^. When the wavelength is tunable within the mid-infrared (MIR) region, namely around 5–10 μm called as a fingerprint region, a variety of physical and chemical processes are expected to occur through an optical vibrational excitation and subsequent energy accumulation in the system, and various researches are reported in medicine, chemical physics, and analytical science^11–13^. In addition to MIR-FEL, far-infrared FEL (FIR-FEL) oscillated at about 50–100 μm that corresponds to approximately 3–6 terahertz (THz) has also been developed at several synchrotron-radiation facilities^14,15^, and the oscillation feature is almost similar with that of MIR-FEL (Fig. 1). Although the application studies using FIR-FEL in biomedical and material engineering fields are limited, the FIR lights exhibit high penetration against the biological substance even under the low radiation energy, and the terahertz lights at several hundred micrometers are strongly absorbed by aqueous matters such as biological tissues^16^. In fact, there have been several successful applications of the far-infrared laser and terahertz spectroscopy to bio-imaging of tissues and low-invasive diagnostics of cancer in medical field^17,18^, and to the molecular study of water in biological physics^19^.

Here, we show that FIR-FEL exhibits remarkable performance in dissociation of fibril structure of a peptide.

## Materials and Methods

### Materials

Five-residue (aspartate-phenylalanine-asparagine-lysine-phenylalanine = DFNKF) peptide (96% purity) was obtained from PH Japan Co. (Hiroshima, Japan). Dimethyl sulfoxide (DMSO), Tris-base, phosphate buffered saline, sodium chloride, and Congo-red were purchased from Wako Pure Chemical Industries (Osaka, Japan).

### FIR-FEL

FIR-FEL developed at The Institute of Scientific and Industrial Research, Osaka University was used in this research (Fig. 1). The FIR-FEL can generate intense pulsed laser beam, and the features are briefly summarized as follows:^20^ (1) oscillation wavelengths are 50–100 μm (**A**). In this study, we prepared four different wavelengths, 60, 74, 80, and 97 μm for the irradiation experiments. The half width was about 5 μm in each spectrum. (2) Time structure is composed of micro- and macro-pulses (**B**): a micro-pulse has a duration of 10 pico-second (ps), and about one hundred micro-pulses are bunched in one macro-pulse with a 4 μs duration. The FEL oscillates at 5 Hz throughout the experiment. (3) Typical pulse energy used was from 0.5 to 1.5 mJ per macro-pulse (**C**).

### MIR-FEL

Detailed oscillation system is described elsewhere^21^. In brief, mid-infrared (MIR)-FEL at Tokyo University of Science was operated at the mid-infrared region (5.0–10 μm: 1,000–2,000 cm^−1^), and the time structure is composed of macro- and micro-pulses, and a repetition rate of macro-pulse is 5 Hz similarly with the case of FIR-FEL, although micro-pulse has a duration of 1 to 2 ps and the interval of the consecutive micro-pulses is 350 ps. The pulse energy per a macro-pulse at around 6 μm was *ca*. 10 mJ.

### Preparation of peptide fibril

The powder of DFNKF was dissolved in DMSO (100 mg/ml) for a stock solution^22^. This solution was diluted by ten times using tris-buffer (20 mM, pH7.5) containing sodium chloride (10 mM) and incubated at 37 °C for several days to produce the fibril. The resulted peptide fibril solution (20 μL) was spread on a stain less steel base to produce a thin film (thickness: ca. 0.1 mm; diameter: ca. 5 mm) for infrared microscopy analysis and on a glass slide base for scanning-electron microscopy observation and Congo-red staining as describe below. After drying under atmosphere for 6 hours, the peptide fibril was irradiated by FIR-FEL or MIR-FEL. The FEL beam was focused on the sample using a parabolic reflector for FIR-FEL or BaF_2_ lens for MIR-FEL. In both cases, the irradiation direction was vertical against the sample surface.

### Terahertz spectroscopy

We used FT/IR-6700FV (Jasco Co., Tokyo, Japan) for measurement of infrared spectrum of peptide at terahertz region. The peptide film was prepared on a stain less steel base as described above, and the measurement was performed in vacuo by reflection mode. As for preparation of the film of pre-fibril, the stock solution of the peptide in DMSO was diluted by the tris-buffer and was immediately dropped onto the stain less steel base at room temperature. The spectrum was recorded with 512 scans.

### Infrared microscopy

The IR spectral measurement was performed using IRT-7000 infrared microscope (Jasco Co, Tokyo, Japan) and FT/IR-6100 spectrometer (Jasco Co., Tokyo, Japan). The infrared spectra were recorded by a reflection mode from several areas on the dry surface of the peptide film with 64 scans and 4 cm^−1^ resolution, and the morphology of the peptide film was observed by 16x Cassegrain lens. The obtained spectral data were transformed into text files, and those absorption intensities at a range of wavenumbers from 1000 to 4000 cm^−1^ were averaged followed by re-converting to infrared spectral trace.

For protein secondary structure analysis, we used IR-SSE analytical software (Jasco Co., Tokyo, Japan). In this program, a calibration curve was prepared prior to multicomponent analysis (Partial Least Squares quantification model) based on the secondary-structural data of 17 proteins, and it was saved as a standard data file^23^. The amide I band was deconvoluted into major four bands: α-helix (1650–55 cm^−1^), β-sheet (1625–40 cm^−1^), β-turn (1655–75 cm^−1^), and non-ordered (other) conformation (1645–50 cm^−1^). Proportions of secondary structures were calculated using peak intensities at those amide-I bands in the averaged IR spectrum.

### Scanning-electron microscopy (SEM)

We used FE-SEM Supra40 scanning electron microscope (Carl Zeiss). After the peptide fibril was added on a glass slide base and irradiated by the FEL as described above, the slide base was fixed on a sample holder by using conductive copper tape, and the surface of the fibrils was observed using the acceleration voltage at 5.0 kV.

### Congo-red staining

The stain solution was prepared by dissolving powder of Congo-red in phosphate-buffered saline to be 0.2 mM concentration. This solution (10 μL) was added on the peptide fibrils on a slide base, and the mixture was incubated for at least 10 min at room temperature. After drying, the surface was observed by using a polarized light microscope MVX 10 (Olympus, Tokyo).

## Results

**Figure 2:**
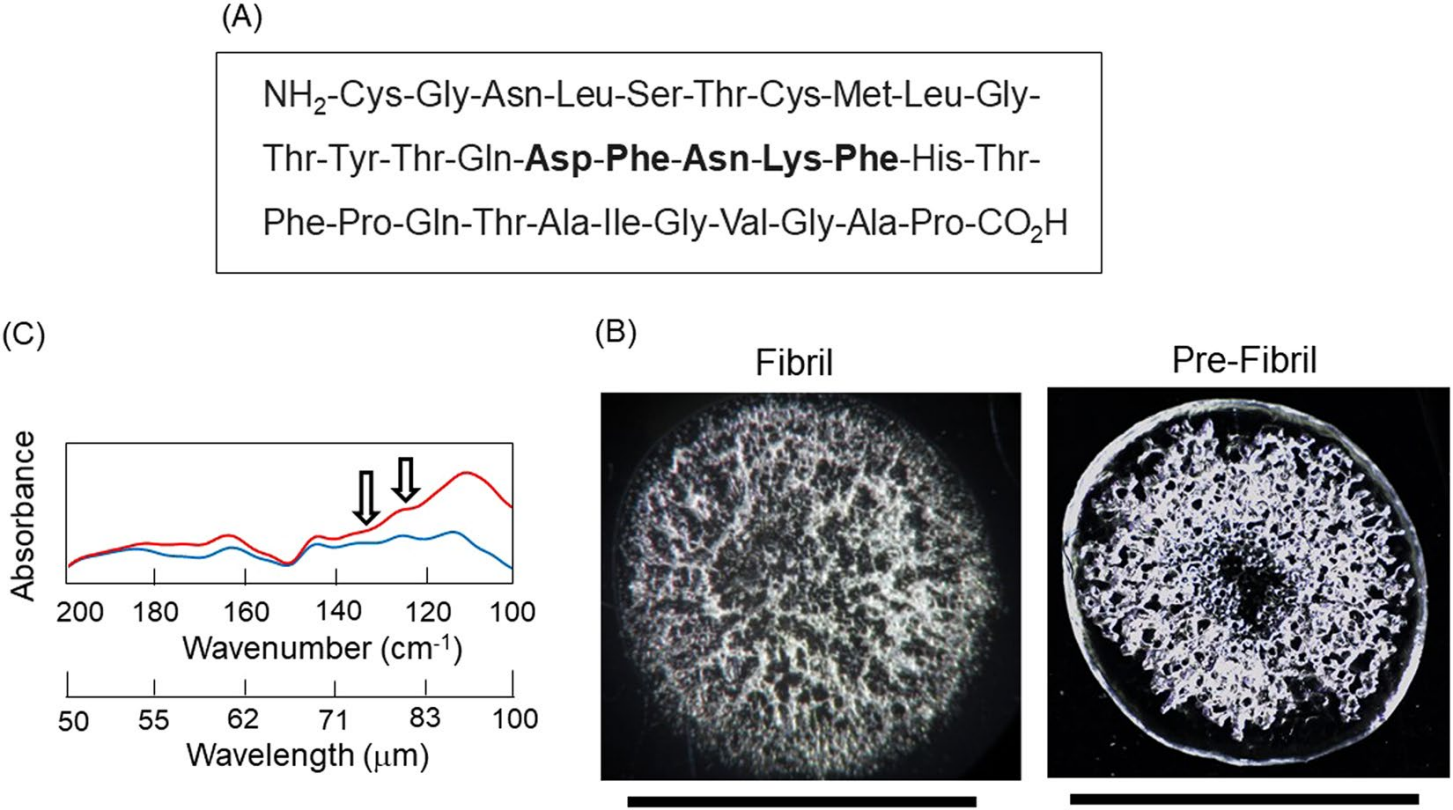
Peptide used the current study and the absorption spectra at terahertz region before and after the fibrillation. (**A**) Amino acid sequence of calcitonin hormone. Five-residue peptide for the fibrillation was indicated in bold. (**B**) Fibril and Pre-Fibril (peptide before fibrillation) dried on a stainless steel base. Black bar: 4 mm. (**C**) Infrared absorption spectra. Red: pre-fibril, blue: fibril. Arrows indicate the irradiation wavelengths (74 and 80 μm) of the FEL.

As a model sample, five-residue peptide (DFNKF) that is a part sequence of calcitonin hormone, was used for the current irradiation experiment (Fig. 2A), because the relationship between its fibrillation and pathogenesis such as medullary thyroid carcinoma is well studied^22,24,25^. It is also established that the internal five-residues used herein readily form fiber-like structure, in which the length of one peptide chain is about several nanometers and a space between the β-strands is about 0.5 nanometers^24^. The peptide fibril and peptide solution before fibrillation (pre-fibril) were dropped onto a steel base (Fig. 2B), and absorption spectra at terahertz region were measured by reflection mode (Fig. 2C). The absorbance of main peaks in the fibril state (blue) was definitely smaller than that in the pre-fibril state (red), although the overall spectral contour in the absorption spectrum was similar. Peptide assembly is generally formed by intermolecular hydrogen bonds between amide groups, and the fibrous peptide becomes more dried than the hydrated state before aggregation. We presume that the reduction of absorbance reflects the loss of water on the occasion of fibrillation, and the higher absorbance for pre-fibril is considered to be due to the decrease of hydrophobicity and hydration of the peptide structure^26^. In the present study, we chose 74 and 80 μm (135 and 125 cm^−1^, respectively) for the irradiation wavelengths, because the oscillation power was comparatively high at those two wavelengths than other wavelengths (60 and 97 μm, Fig. 1C).

### Reduction of β-sheet conformation by the terahertz radiation

**Figure 3:**
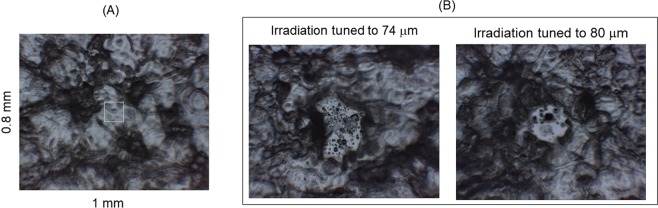
Surface observation of peptide film before and after irradiation. (**A**) Peptide fibril before irradiation. White square: aperture size (100•100 μm) for infrared microscopy. (**B**) Peptide fibrils after irradiations at two wavelengths. Scales were the same with those in (**A**).

**Figure 4:**
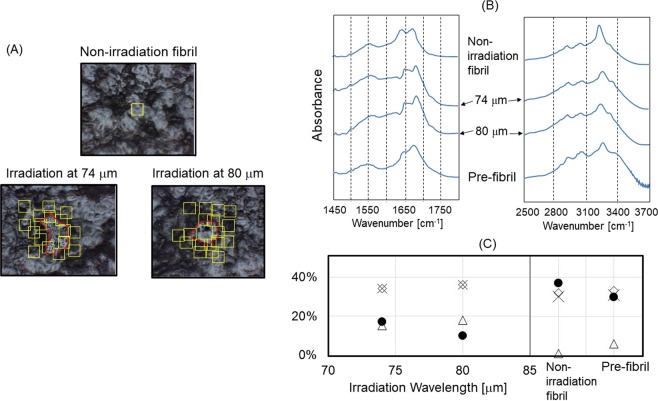
Infrared microscopy analysis. (**A**) Analytical area on the peptide film. Visual fields for recording the infrared spectra around the irradiated spot (red circle) were shown as yellow squares (100•100 μm). For the peptide fibril before irradiation (Non-irradiation fibril), a representative area was shown. (**B**) Averaged spectra at mid-infrared region (left) and at near-infrared region (right). Infrared spectrum before fibrillation (Pre-fibril) was obtained as single spectrum in the transmission mode by using potassium bromide pellet. (**C**) Conformational analysis. The proportion of each conformation (α-Helix [triangle], β-Sheet [black circle], β-Turn [cross], and other conformation [diamond]) was obtained based on the intensity at amide I band in the averaged spectrum (**B**).

Both of irradiations gave visible damage on the peptide film after 30 min irradiation (~9000 pulses) with *ca*. 1 mJ energy per a macro-pulse: small holes were obviously observed (Fig. 3B) compared to the fibril before irradiation (Fig. 3A). The sizes of these spots were ~200–300 μm in diameter that corresponds to the focused beam diameter, in which a clear circle was seen in case of irradiation at 80 μm compared to that at 74 μm. The infrared absorption spectra, recorded from several areas as shown by yellow squares in the surrounding debris (Fig. 4A), were averaged and compared before and after the irradiation (Fig. 4B, see Supplementary Materials). Doublet structure of the amide I peaks from 1600 to 1700 cm^−1^ are common spectral features of the peptide in the mid-infrared region (left panel). Reches *et al*.^22^ attributed the amide I peak at lower wavenumber (~1645 cm^−1^) to the C=O stretching vibrational mode of amide bonds in β-sheet stacked structure, because (i) the peak intensity at ~1645 cm^−1^ is weaker than that at higher wavenumber (~1670 cm^−1^) before fibrillation (Pre-fibril) and (ii) the former intensity increased after fibrillation (Non-irradiation fibril). It should be noted that the peak intensity at higher wavenumber increased after irradiation at 74 and 80 μm, and the double peaks were slightly shifted to higher wavenumbers (plus ca. 10 cm^−1^) after those irradiations, while amide II band (at ~1550 cm^−1^) and a tiny peak at 1500 cm^−1^ little moved. These spectra show that conformations regulated by the amide backbone in the fibril state were changed by the irradiation, and the blue shift of the amide I bands and an increase of peak intensity at higher wavenumber (~1670 cm^−1^) may indicate that rigid conformation was raveled out to the non-aggregate form.

In the near infrared region (right panel), the O–H stretching vibrational band was observed at around 3250 cm^−1^. The sharp peak was observed for the fibril state (top), and the broadened band with shoulder at 3300–3400 cm^−1^ was characteristic for the pre-fibril state (bottom). Interestingly, irradiations at 74 and 80 μm to the fibril state afforded the similar spectra as that of the pre-fibril state: the peak at 3250 cm^−1^ was broadened and a shoulder peak at ~3300 cm^−1^ was clearly seen. These near-infrared spectra support FIR-FEL driven conformational change in the fibril structure at specific far-infrared wavelengths. Together with the spectral change in the amide I region, the FEL irradiation may disrupt hydrogen bonds between β-sheets in the fibril state and produce non-aggregated free peptide chains.

To estimate the conformational change of the peptide fibril in more detail, the ratio of four types of secondary conformations (α-helix, β-sheet, β-turn, and other) were derived through the spectral deconvolution of the amide I band (Fig. 4C, see also Supplementary Materials). For non-irradiation fibril (right panel), the content of β-sheet (black circle) was ~40%, those of β-turn (cross) and other conformations (diamond) were ~30% each, and α-helix (triangle) was hardly detected. For pre-fibril state, α-helix was recognized (~5%), and the β-sheet content was slightly lower than that of non-irradiation fibril state. In contrast, both irradiations at 74 and 80 μm substantially reduced the amount of β-sheet down to about 10‒20% and increased that of α-helix to near 20% (left panel). Interestingly, the amount of β-sheet was lower and that of α-helix was higher in both cases than those in the pre-fibril state. In addition, it seems that the irradiation at 80 μm reduces the β-sheet conformation in the fibril more remarkably than that at 74 μm.

### Comparing with mid-infrared FEL (MIR-FEL) irradiation and thermal treatment

**Figure 5:**
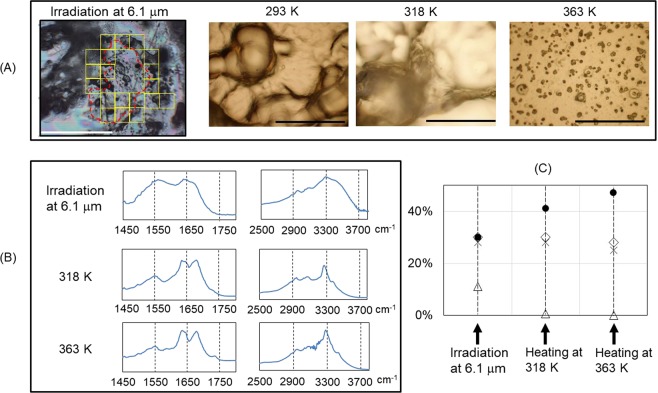
MIR-FEL irradiation and thermal processing. (**A**) Infrared microscopy observation after the FEL irradiation at 6.1 μm (left), optical microscope image of the fibril on gold-coated glass slide before heating (293 K), and those after heating (318 K and 363 K). White bar: 500 μm; black bar: 125 μm. The irradiated position was indicated by red dotted line, and visual fields for collecting infrared spectra were shown as nineteen yellow squares (100•100 μm each). (**B**) Averaged spectra of fibrils after irradiation (upper), heating at 318 K (middle), and heating at 363 K (bottom). For the heated samples, about 20 spectra were recorded using 20•20 μm apertures and averaged. (**C**) Conformational analysis. Labels were the same with those in Fig. 4C.

To ascertain the peculiarity of FIR-FEL on the peptide fibril, we examined MIR-FEL irradiation tuned to 6.1 μm, because we observed that amyloid β fibril was dissociated to the non-aggregate form by the MIR-FEL at amide I band in a previous study^27^. The shape of the irradiated position on the peptide film was fuzzy (red trace in Fig. 5A), but infrared spectra could be recorded from several areas (yellow squares) in the surrounding region (Fig. 5B, upper, see also Supplementary Materials). The double amide I bands at around 1650 cm^−1^ were broadened, and peak around 3400 cm^−1^ was largely increased, compared to those in the non-irradiation fibril (Fig. 4B). Nonetheless, ratio of α-helix and β-sheet could be calculated to 11% and 30%, respectively (Fig. 5C). Irradiation time was 30 min similarly with the FIR-FEL, and the irradiation power was set to 5.6 mJ per macro-pulse in case of MIR-FEL. These results indicate that the MIR-FEL certainly changed the conformation of the fibril of the DFNKF peptide, but its dissociative effect was not so remarkable compared to that by the FIR-FEL at 74 and 80 μm (Fig. 4C), even though the macro-pulse energy in the former was higher than that in the latter (~1 mJ).

Furthermore, we compared the FEL irradiation with thermal treatment. In case of heating, the morphology of the peptide was absolutely destroyed, and its surface was solidified and flat at 363 K, while it was observed as uneven soil at 293 and 318 K (Fig. 5A). Infrared microscopy analysis showed that the double amide I bands at 1645 and 1670 cm^−1^ were little shifted and a peak intensity at 3300 cm^−1^ was elevated by increasing the temperature to 363 K, compared to that at 318 K (Fig. 5B, middle and bottom). Interestingly, secondary structure analysis showed that the heating rather increased the β-sheet content to near 50% and little changed the α-helix content (~0%) (Fig. 5C). Therefore, thermal treatment did not reduce fibril proportion but increased the fibrous feature.

### Laser-power dependent dissociation of peptide fibril

**Figure 6:**
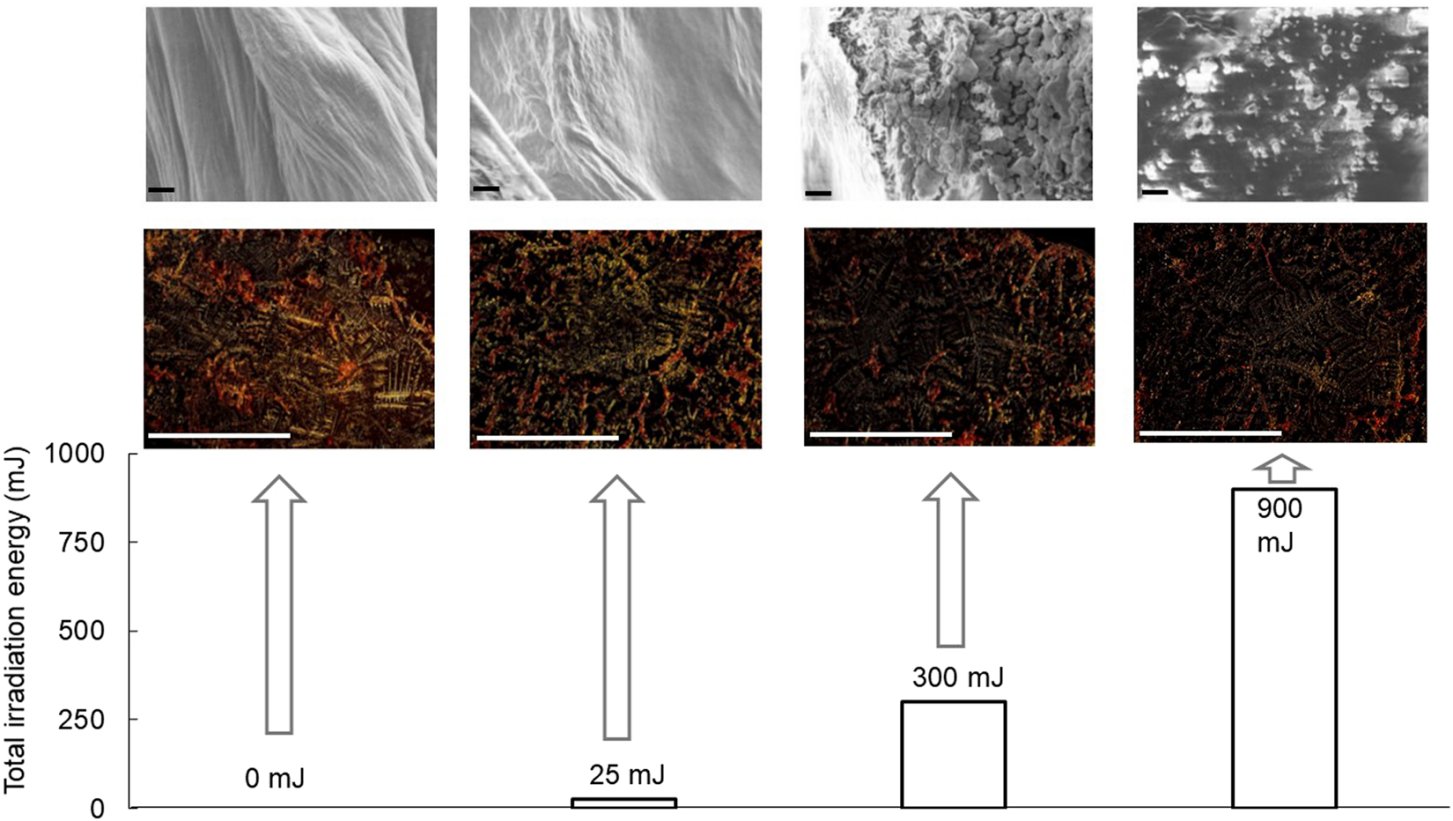
Irradiation-power dependent morphological evolution of peptide fibril. Vertical axis: total power energy (mJ) supplied with the FEL. Upper: SEM images, lower: Congo-red staining images. Black bar: 1 μm; white bar: 500 μm.

Finally, morphological change of the peptide induced by the FEL irradiation was investigated using scanning-electron microscopy (SEM) and Congo-red staining (Fig. 6). In the non-irradiation fibril, soft cloth-like bundles made from many fibers were observed in places of the surface of peptide film, in which the length of the fiber was more than 10 μm. When the wavelength of FEL was tuned to 74 μm and the total irradiation energy reached to 25 mJ (equivalent to 25 shots with 1 mJ energy per macro-pulse), a slight change on the surface morphology was observed. The fibril bundles were visibly destroyed at 300 mJ (3000 shots with 0.1 mJ per macro-pulse) and were completely dispersed at 900 mJ (3000 shots with 0.3 mJ per macro-pulse). Congo-red staining also showed a laser-power dependent dissociation of the fibril; the yellow-green brightness of birefringence due to binding of the reagent into the β-sheet backbone decreased as the irradiation energy increased.

## Discussion

The self-associated peptides such as amyloid fibrils not only work as pathological cause but also have roles for maintaining life activities and for expression of biological function^4^. In eukaryotic cells, those biological functions are cooperated by many structural proteins such as collagen, keratin, and actin, and these structural proteins construct rigid peptide networks for maintaining cytoskeleton^28,29^. The structural regulation method of those peptide assemblies by using external physical forces such as electromagnetic waves has a potential for changing the biological functions associated with the filamentous network. Previously, MIR-FEL was originally applied for dissociation of amyloid fibrils for the purpose of development of novel physical engineering technique for the disease therapy, and we found that the MIR-FEL tuned to wavelengths 6.1–6.2 μm can dissociate the aggregate structure of amyloid peptide into the non-aggregate form^27^. In the current study, we found that the FIR-FEL irradiation tuned to 70–80 μm changed the fibril state of a peptide more remarkably than the MIR-FEL, as shown in the conformational changes of peptide. Although it cannot be disclosed the reason why the far-infrared wavelength was different from the mid-infrared wavelength in the point of reduction of the fibrous conformation of peptide, the terahertz wave can penetrate to the depth of the thick material, and it can be considered that the FIR radiation disrupts hydrogen-bond network inside of the aggregate structure more effectively than the MIR-FEL. In case of MIR-FEL irradiation at about 6 μm, the trigger of fibril dissociation is vibrational excitation of amide bonds, and only a part of the surface area of the peptide film is influenced by the mid-infrared radiation. Therefore, we presume that the difference in the energy permeability of the FEL between mid- and far-infrared regions will be appeared in difference of the dissociative effect on the peptide fibril. From this point of view, it can be expected that the terahertz FEL would be employed for reducing the amyloid fibrils that are deposited in internal tissues, while the MIR-FEL would be effective for dermal amyloid present in the upper part of skin. A possible challenge is introducing the terahertz FEL into the amyloid-rich tissues to test if the laser is applicable for selective reduction of amyloid fibrils in biological systems. For example, application of the FIR-FEL to dissolution of amyloid β fibril in brain section of Alzheimer’s disease model mouse can be proposed in future. Another valuable use of the terahertz FEL is the application as fabrication tool for biomaterial. Peptide fibril can be used as fiber-type scaffold for biocompatible materials such as cell sheets in regenerative medicine and nano-carrier in drug-delivery system^30,31^. Intense terahertz laser would be expected to become a novel processing tool of those biomaterials both *in vivo* and *in vitro*. As described above, the dissociative effect on the peptide fibril by FIR-FEL is basically similar with that by MIR-FEL: β-sheet conformation was reduced by the intense infrared laser. We have already found that several types of amyloid fibrils such as inulin, amyloid β, and polyglutamine were similarly dissociated by the MIR-FEL^32,33^, and so it is very interesting how FIR-FEL would process various types of peptide fibrils. This is a next research project.

In summary, we tested the FIR-FEL at terahertz wavelengths from 70 to 80 μm to irradiate fiber-like peptide aggregate. The conformational analysis based on the amide I regional spectra showed that β-sheet was reduced and α-helix was increased by the FIR-FEL more remarkably than the MIR-FEL tuned to 6.1 μm, and the dissociative effect by the FEL was contrast with thermal effect with regards to changes in the fibrous conformation. In addition, the fibril state was laser-power dependently from 25 to 900 mJ energy dissociated at micro scale. This study showed an impact of FIR-FEL irradiation on the biomolecular aggregate and may open a new way of intense terahertz laser as processing tool of fibrous peptide in medical and material engineering fields.

## Supplementary information

Supplementary Information for Dissolution of a fibrous peptide by terahertz free electron laser

FTIRdata20190515

Conformation Analysis20190515

## Data Availability

A part of data is represented as Supplementary Materials, and other experimental data are available from the corresponding author (T.K.). The Supplementary Materials are available in the Supplementary Information.
