# Supplementary material for: Dissolution of a fibrous peptide by terahertz free electron laser
Source: Sci Rep. 2019 Jul 23;9:10636. doi: 10.1038/s41598-019-47011-z (PMC6650392; doi:10.1038/s41598-019-47011-z)
Supplement: Supplementary file 1 — Supplementary Information for Dissolution of a fibrous peptide by terahertz free electron laser [file 41598_2019_47011_MOESM1_ESM.doc]

**Supplementary Information**

for

**Dissolution of a fibrous peptide by terahertz free electron laser**

Takayasu Kawasaki^1^*, Koichi Tsukiyama^1^, and Akinori Irizawa^2^

^1^IR Free Electron Laser Research Center, Tokyo University of Science, 2641 Yamazaki, Noda, Chiba 278-8510, Japan.

^2^ The Institute of Scientific and Industrial Research, Osaka University, Mihogaoka 8-1, Ibaraki, Osaka 567-0047, Japan.

*Correspondence to: kawasaki@rs.tus.ac.jp

1. **FT-IR data**

FT-IR spectrum of peptide was measured using infrared microscopy IRT-7000 (Jasco Co., Tokyo, Japan) combined with Fourier Transform Infrared Spectrometer FT/IR-6100 (Jasco Co., Tokyo, Japan). The peptide fibril was dropped onto the flat metal base (20 μL solution) and dried under atmosphere. After the FEL irradiation, several regions that were separated by a square 100 μm on a side were selected around the irradiated spot, and the IR spectrum was recorded by reflection mode using 64 scans at a 4 cm^-1^ resolution from 500 to 4000 cm^−1^ wavenumbers. All raw IR absorption data were uploaded on an Excel file: file name# **FTIRdata20190515**. This file contains 7 sheets and each sheet name is as follows: Non-irradiation fibril, Irradiation at 74 μm, Irradiation at 80 μm, Pre-fibril, Irradiation at 6.1 μm, Heating at 318 K, and Heating at 363 K.

1. **Conformational analysis data**

The four conformations (α-helix, β-sheet, β-turn, and non-ordered (other)) were analysed based on the peak intensity of the amide I band of the peptide. Those proportions (%) were calculated by using software IR-SSE (Jasco Co., Tokyo, Japan) and described in an Excel file: file name# **Conformation Analysis20190515**. All data were represented as averaged values except for fibril before fibrillation (named as Pre-fibril). In cases of fibril before irradiation (named as Non-irradiation fibril), fibril after irradiation at 74 μm (Irradiation at 74 μm), and fibril after irradiation at 80 μm (Irradiation at 80 μm), the calculation of the conformational proportion was performed using the averaged IR spectrum (Supplementary file **FTIRdata20190515**). In case of Pre-fibril, single spectrum was obtained by transmission mode using KBr. In cases of the fibril after irradiation at 6.1 μm (named as Irradiation at 6.1 μm), fibril after heating at 318 K (Heating at 318 K), and fibril after heating at 363 K (Heating at 363 K), several IR spectra were recorded, and the proportions of four conformations were calculated at each spectrum followed by averaging. Standard deviation was also added with the averaged value at each data sheet.
